# Positive mental health in psychotherapy: a qualitative study from psychotherapists’ perspectives

**DOI:** 10.1186/s40359-022-00816-6

**Published:** 2022-04-29

**Authors:** Sherilyn Chang, Rajeswari Sambasivam, Esmond Seow, Mythily Subramaniam, Hanita Ashok Assudani, Geoffrey Chern-Yee Tan, Sharon Huixian Lu, Janhavi Ajit Vaingankar

**Affiliations:** 1 Research Division, Institute of Mental Health, Singapore, Singapore; 2 Department of Psychology, Institute of Mental Health, Singapore, Singapore; 3 Department of Mood and Anxiety, Institute of Mental Health, Singapore, Singapore; 4 Singapore Institute of Clinical Sciences, A*STAR, Singapore, Singapore

**Keywords:** Positive mental health, Wellbeing, Psychotherapy, Qualitative study

## Abstract

**Background:**

There is growing evidence in the literature on the use of positive mental health (PMH) interventions among clinical samples. This qualitative study aims to explore the definitions of PMH from psychotherapists’ perspectives, and to examine views and attitudes related to the construct.

**Methods:**

Focus group discussions were conducted with psychotherapists at a tertiary psychiatric institute. Focus group sessions were transcribed verbatim and transcripts were analyzed using an inductive thematic approach.

**Results:**

Five themes related to psychotherapists’ definition of PMH were identified: (1) acceptance; (2) normal functioning and thriving in life; (3) resilience; (4) positive overall evaluation of life; (5) absence of negative emotions and presence of positive emotion states. Themes related to views and attitudes towards PMH were: (1) novel and valuable for psychotherapy; (2) reservations with terminology; (3) factors influencing PMH.

**Conclusion:**

PMH in psychotherapy is a multidimensional concept that means more than symptom management and distress reduction in clients. There is potential value for its application in psychotherapy practice, though some concerns need to be addressed before it can be well integrated.

**Supplementary Information:**

The online version contains supplementary material available at 10.1186/s40359-022-00816-6.

## Introduction

Positive mental health (PMH) reflects a state of mental wellbeing that goes beyond the mere absence of psychopathology. It encompasses emotional and psychological wellbeing, and functioning in psychological, social and societal domains [1]. In relation to emotional wellbeing, Diener et al.’s definition on subjective wellbeing is often drawn upon as it looks at an individual’s overall evaluation of their life and emotional experiences such as life satisfaction, positive affect and negative affect [2]. This is also seen as the hedonic approach to wellbeing that focuses on pleasure attainment and pain avoidance [3]. In contrast, the eudemonic approach ‘focuses on meaning and self-realization and defines wellbeing in terms of the degree to which a person is fully functioning’ [4]. A closely related concept of psychological wellbeing has been conceptualized as consisting of six dimensions: autonomy, environmental mastery, personal growth, purpose in life, positive relations with others, and self-acceptance [5]. In addition to emotional and psychological wellbeing, Keyes also considers social wellbeing as essential in identifying a thriving individual as flourishing [6]. Some studies have also identified spirituality (related to religious beliefs and practices) as an important domain of PMH [7, 8].

Traditionally psychotherapy, and also clinical care in general, focus on alleviating symptoms and are largely aimed at correcting deficits resulting from disruption of normal functioning [9, 10]. There have been calls to shift away from this deficit-based view of mental health to promoting wellbeing, and to evaluate both PMH dimensions and psychopathology when providing psychotherapy and in conducting research [1, 11, 12]. Distinct psychotherapeutic interventions that are theoretically grounded in positive psychology, a scientific field that studies contributing factors of human flourishing and optimal function [13], explicitly targets wellbeing outcomes. Some examples include therapies such as the wellbeing therapy [14] and positive psychotherapy [15]. Other non-positive psychology approaches such as mindfulness-based interventions and gratitude-promoting exercises have also been incorporated into traditional psychotherapies to enhance wellbeing.

Several studies have examined the effectiveness of PMH interventions in improving outcomes among clinical samples. A meta-analysis by Goldberg et al. reported equivalent efficacy of mindfulness-based interventions to first-line, evidence-based psychological and psychiatric treatments in symptoms reduction, with effects most consistent for depression, pain, smoking and addictions [16]. In another meta-analysis examining the effects of positive psychology interventions on wellbeing and distress, the authors found small effect sizes for such interventions on improving wellbeing and depression, and moderate improvements for anxiety among clinical samples [17]. A recently published article by Jankowski et al. provided a comprehensive review of the various types of interventions in psychotherapy to promote wellbeing and the efficacy of these treatments [11]. The authors found support for these approaches in enhancing wellbeing and urged ‘researchers and psychotherapists to continue to integrate symptom reduction and wellbeing promotion into psychotherapy approaches aimed at fostering client flourishing’. Given that ‘good outcomes’ of psychotherapy constituted more than symptom alleviation and included outcomes such as with gaining acceptance and self-understanding, alongside developing a sense of mastery and self-compassion [18], there is value in exploring the application of PMH interventions in psychotherapy.

To date there has been no study that has examined the concept of PMH among psychotherapists and to understand their attitudes towards a PMH based approach in psychotherapy. As a step towards exploring ways in which PMH interventions can be incorporated into psychotherapy practice, it is imperative to first understand the concept of PMH from the point of view of psychotherapists in clinical settings. This could provide insights into the attitudes of psychotherapists towards PMH and identify potential challenges and difficulties in integrating PMH interventions into psychotherapy in a clinical setting. The present qualitative study thus aims to explore the definitions of PMH from psychotherapists’ perspectives and its application in their practice, and to examine their views related to the concept of PMH.

## Methods

### Study design and setting

A qualitative study was conducted at a tertiary psychiatric hospital in Singapore and data collection took place between April and November 2019. This study used an interpretivist approach to gain an in-depth understanding of psychotherapists’ definitions and views of PMH, and this enabled an understanding of psychotherapy practices from the practitioners’ perspectives to yield clinical applications and inform future research. The Consolidated Criteria for Reporting Qualitative Research (COREQ; Additional file 1: Appendix A) was used to guide the reporting of this study [19].

### Study sample

Participants for this study were professionals who provided psychotherapy to individuals with mental health issues at private or public institutions in Singapore. Purposive sampling was adopted to ensure appropriate representation of psychotherapists by work experience. Psychotherapists were invited to participate in the research study through connections from personal network and also via word of mouth (none of the recruited participants were personally acquainted with study team members who were present during the interview), and were contacted through phone calls and emails to provide them with further details of the study. Inclusion criteria for the study were individuals aged 21 years and above, experienced in providing psychotherapy to people with mental health problems at public or private institutes, and able to provide consent. The study was approved by the institutional ethics committee and all participants had provided written informed consent prior to their participation. This study was conducted in accordance with the Declaration of Helsinki.

### Procedure

Qualitative data was collected during focus group discussions (FGDs) conducted with psychotherapists. Each FGD session lasted between 1.5–2 h, had 4–6 participants, and was facilitated by a female senior researcher (JV), who has a background in epidemiology (MSc) and is trained in qualitative research methodologies and has domain expertise in the area of mental wellbeing. Study team members (RS, ES or SC), who were researchers with bachelor degrees in psychology and had prior experience in conducting qualitative research, were present during the session as a note taker. Participants completed a short questionnaire that collected information pertaining to their sociodemographic background and clinical experience. As part of icebreaking activity before the FGD began, all participants and study members who were present briefly introduced themselves regarding their work and personal interests. An interview guide was used during the FGDs to facilitate discussion (see Table 1 for brief guide). This interview guide was developed with inputs from clinicians and psychologists from the study team to set the questions in the context of psychotherapy. Participants were first presented with an overarching question on what PMH means to them in their practice, and were then given time to pen down their thoughts on cue cards. These cards served as aids to facilitate subsequent discussion. As far as possible, the discussions followed the experiences of the participants and clarifications were sought when needed. Participants were also encouraged to share their opinions on the viewpoints raised by other participants during the discussions. Recruitment of participants and FGDs continued until repetition of themes occurred and no new information was evident (i.e. data saturation achieved). All the FGDs were audiotaped and transcribed verbatim for analysis. Quality checks on the transcripts were performed; after which the transcripts were anonymized to safeguard the participants’ identity.

**Table 1:** Brief guide of questions and probes used

*Definitions of Positive Mental Health (PMH)* What does PMH mean to you in psychotherapy practice? When you say [construct], what are the things that you are thinking of? Can you give me some examples? How do you apply that in your practice? How would you define [construct]? Why do you think [construct] is PMH? How do you think [construct] helps in PMH? *Factors influencing PMH* What are the factors that promote PMH or psychological wellbeing? How do you think that would be a factor in a person’s mental health?	

### Analysis

Thematic data analysis was conducted to analyze the data where common underlying themes were identified inductively from the data [20]. NVivo software (Version 11) was used to code and organize the data. One transcript each was assigned to three study team members (JV, SC, ES) who read through the respective transcript repeatedly and thoroughly to familiarize themselves with the content. Each team member noted meaningful content in the transcript to generate codes inductively which were later combined to form emergent themes. Study team members then gathered to discuss the codes and themes obtained, and a list of preliminary themes was identified. This was used to code the remaining transcripts, and new codes and themes were created to capture any new content that emerged. After all transcripts were reviewed, various themes were combined to produce higher-order themes. Any disagreements between team members were resolved through discussions to reach consensus.

Lincoln and Guba’s criteria to assess the trustworthiness of a study looks at credibility, transferability, dependability and confirmability [21], and these criteria can be applied in conducting thematic analysis [22]. In terms of data accuracy, all FGD sessions were audio-recorded and transcribed verbatim by a team member; study team members (other than the person who transcribed the interview) performed checks on the transcripts to ensure its quality and accuracy. Raw audio recordings and verbatim transcripts were stored in well-organized archives until verification was completed, and records of observation notes, coded transcripts and discussion notes were kept to provide an audit trail of the code generation process and serves to provide dependability and confirmability. Findings were reviewed by members in the study team which included researchers and also psychotherapists and this addresses credibility of the study. Detailed descriptions of the research process and in reporting of results can provide information to other researchers on the transferability of findings in another study population.

## Results

A total of 7 FGDs were conducted with 38 participants for the study. The participants’ age ranged between 27 and 63 years, were mostly females (84.2%), of Chinese ethnicity (81.6%), and the majority had obtained a post-graduate degree (94.7%; Table 2). All participants had received formal training in varied psychotherapy modalities including cognitive behavioral therapy, positive behavioral management, exposure and response prevention, eye movement desensitization and reprocessing, acceptance and commitment therapy, schema-focused therapy, emotion focused therapy, solution focused brief therapy, psychodynamic therapy, dialectical behavioral therapy, mindfulness-based therapy etc. For the majority of participants, their clientele comprised adults presenting with mental disorders including mood disorders and anxiety disorders. Others worked with children and adolescents with childhood disorders, elderly population with dementia, or individuals who needed life coaching.

**Table 2:** Sociodemographic profile of participants (n = 38)

	n
Age (mean)	35.7
*Gender*	
Female	32
Male	6
*Ethnicity*	
Chinese	31
Indian	4
Others	3
*Highest education*	
University degree	2
Postgraduate degree	36
*Employment setting*	
Public institution	34
Private practice	3
Both	1

**Fig. 1:**
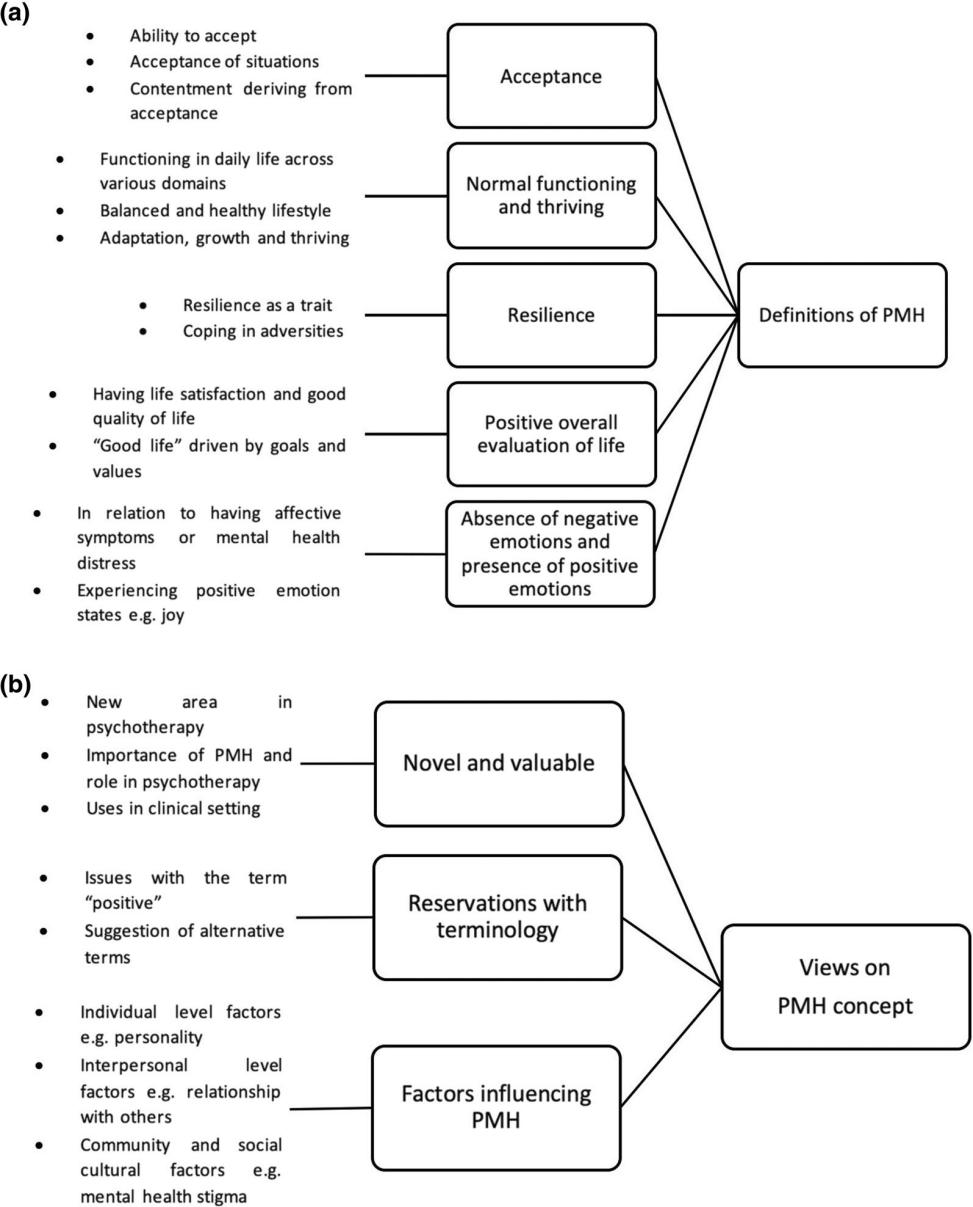
**a** Coding tree of themes identified in the coding process pertaining to psychotherapists’ definition of PMH. **b** Coding tree of themes identified in the coding process pertaining to psychotherapists’ views on the concept of PMH

Thematic analysis of the qualitative data identified five themes pertaining to psychotherapists’ definition of PMH: (1) acceptance; (2) normal functioning and thriving in life; (3) resilience; (4) positive overall evaluation of life; (5) absence of negative emotions and presence of positive emotion states. Their views on the concept of PMH could be examined from the following three themes: (1) novel and valuable for psychotherapy; (2) reservations with terminology; (3) factors influencing PMH. Figure 1a, b present the coding trees derived from the coding process with the subthemes and themes shown. The following section describes the themes in further details and salient quotes that underscore the essence of the theme are presented.

### Definitions of positive mental health

(1) Acceptance

This was a common theme discussed by participants from various FGDs. PMH was defined as having the ability to accept things that happened in life and acknowledging the outcomes that resulted. Acceptance was in relation to not just negative events, but also acceptance of ‘difficult emotions’ and where one is in life.

It’s about accepting where you are in life and… as well as… growing in that journey to acceptance and being at peace with that. – FGD 3

Same way like you were talking about ACT (Acceptance and Commitment Therapy) just now, it’s accepting it, even if they just accept what has happened to them, I think it’s already positive mental health. – FGD 7

In a related note, a participant described PMH as having contentment in life and described how acceptance of situation contributed to contentment.

Positive mental health to me is finding content, which is a bit like peace, whatever the circumstance… a lot of it is perception, how you see certain things, like certain circumstances that you might not be able to control. So I mean modifying or coming to terms with what I can accept and what I can change. I think that helps; gives me contentment and peace. – FGD 6

(2) Normal functioning and thriving in life

For the participants, having PMH was defined as being able to function normally. At the individual level, a functioning person was described as someone leading a balanced and healthy lifestyle, and able to manage stress and not be overwhelmed by it. The idea of optimal functioning pertained to various aspects in life including occupation, relationship with others, and being an active and contributing member of the society.

PMH is not about like the mental condition. It is about, you know, how we make these conditions and maybe other life issues not to interfere with our life. So it’s about living that life, you know, despite all the obstacles and difficulties. – FGD 3

Some participants moved beyond the notion of basic psychosocial functioning to describe PMH in terms of thriving which encompassed the idea of growth.

I wrote it (PMH) as the ability to thrive in very stressful environment [be]cause I think the way I see PMH is not just the absence of mental health issues but [it] is also the ability to kind of progress and really to be able to kind of expand on your own potential. – FGD 4

… they (clients) are kind of bootstrapped. They are self-corrective. They may come to you with a presenting problem, but if you just drop a few hints along the way, a bit of psycho-edu[cation] here, a bit of coaching there, they are able to extrapolate that to other problems independently on their own. So I think that’s also important. It’s not just where you are now, it’s whether you have the capacity to adapt and grow. – FGD 6

(3) Resilience

In defining PMH, the concept of resilience was frequently brought up by participants and it at times co-occurred alongside the theme on functioning. Yet this is a distinct theme from functioning in that rather than focusing on outcomes, it describes a trait or skillset that promotes wellbeing.

I would see it (PMH) as resilience, the ability to deal with challenges and the ability to function. – FGD 1

Okay for me positive mental health is being able to cope with the demands and challenges of life. So it’s a bit like mental resilience… sometimes you have negative emotions and being able to cope with that or cope with the demands. – FGD 2

Resilience was often described by participants as a trait that would help their clients to ‘bounce back’ from adversities, and also as a coping resource to support normal functioning in spite of challenges. One participant discussed how having emotional resilience can aid distressed clients to self-regulate by learning to not internalize events that occurred around them.

(4) Positive overall evaluation of life

The keywords in this theme were ‘quality of life’, ‘good life’, ‘fulfilled life’ and ‘life satisfaction’. Definitions captured in this theme described the concept of PMH as an all-encompassing, overall evaluation of one’s life that generated a broad sense of wellness or a feeling of ‘good living’.

Good living, like you’re not just alive; but you are living well, so living well… I think it’s defined differently by different people. So to person A living well might be ‘I’m a able to look after my grandkids’, that’s living well… to summarize it’s the person’s own idea of a good life, a good quality of life. – FGD 1

While elaborating the concepts of life fulfillment and the ideal life in the context of PMH, keywords such as ‘goals’, ‘values’, ‘purpose’, ‘meaningful’ and ‘aspirations’ were often mentioned and participants described these as constituents of a ‘good life’.

… a feeling of living a life that is consistent with one’s values… If someone values career, then he is living a life that is working towards that. If my value is family, I’m living a life that allows me to spend time with my family in a way that I consider meaningful. – FGD 5

Positive mental health is leveraging on people’s needs and values to bring them closer to their fulfilment… To me, fulfilment is living their own values, living their lives according to their own values. And being able to meet their needs. – FGD 6

(5) Absence of negative emotions and presence of positive emotion states

This theme relates to the emotional state of an individual and the definitions of PMH encompassed the absence of distress and the presence of positive emotions. PMH was defined as the removal of mental illness symptoms or distress, and also it meant experiencing positive emotions and state such as ‘happiness’, ‘hope’ and ‘joy’.

Freedom in mind, having peace, having calm. And there is no mental illness or distress and managing with difficulties. – FGD 4

I’ve written that firstly, positive mental health is being hopeful and laughing often. – FGD 5

### Views on the concept of PMH

(1) Novel and valuable for psychotherapy

For some participants, PMH was a novel concept which could be defined in various ways by different individuals. For one participant, it gave the ‘impression of mindfulness’ which is the ‘third wave of therapy at the moment’, and some participants compared it to positive psychology.

So I think positive mental health is a new change, so it’s like a new science where you hear a lot of people saying that oh it’s important, it’s crucial but the research out there is very limited to back up all this evidence, but we do see the trends of positive mental health is emerging too. – FGD 2

Participants generally agreed on the importance of individuals to have PMH, with one participant stating it as ‘our birth right’, and another participant citing it to be ‘imperative for a healthy society’. A number of participants acknowledged the roles that they could potentially play as psychotherapists in introducing PMH concepts to their clients, as evident from the following quotes:

Like traditionally the way therapy was created was for like to remove disorder. That’s why I think the newer age therapists are saying that we really need to go further where there’s this idea of growth. I think that’s where the newer age therapists try to incorporate it as part of therapy. – FGD 4

I think for me they (PMH-based interventions) definitely have a space in psychotherapy and they help to balance out between always talking about problems as compared to, well, talking about what were you like before all the problems and what would it be like without the problems. So it balances out the conversation a little bit as compared to every time you come in we talk about your difficulties. – FGD 5

Not all participants, however, concurred with the relevance and significance of PMH, particularly in the context of clinical setting and the profile of clientele that they saw.

I think positive psychology is not that much used in our setting maybe because we have quite a lot of patients in quite severe conditions and talking about positive psychology is like… we are at this level and then you are talking about positive psychology. So maybe in our setting, clinical setting, we don’t really talk about positive psychology and I find that it’s more of a marketing thing… like it’s great and we are doing these classes in school and all that but I think there are other things that are more important to be done. – FGD 2

(2) Reservations with the terminology

A number of participants expressed reservations with the term ‘positive’ that was being used, either with respect to ‘positive mental health’ or ‘positive psychology’. To some participants, such usage implied that clients have to strive towards a positive state all the time, which is ‘not natural’ and ‘an impossible setup’ for them, when instead a simple improvement or progression could in fact be thought of as ‘positive’.

Because from clinical psychology background, it’s about treating mental illness. So it’s like if they (clients) can reach a neutral level or it may be back to baseline, then it’s something the patient may know to achieve, so positive means it sounds to me like up there (pointing to higher level). That you know even myself cannot be completely happy all the time. – FGD 3

The word ‘positive’ here is very misleading. And it’s exaggerating people’s expectations… it’s like wherever you are, if things get in anyway slightly better… that’s already positive. It need not necessary be like you have to have ten steps of growth, not really. – FGD 7

Some participants felt that this terminology carries a connotation and dichotomizes mental health either into the positive or negative realm, and that did not accurately reflect the entirety of what mental health should be in their psychotherapy practice.

I guess one of the main core tenets of psychotherapy is to bring flexibility and balance in the ideas or the perspective that we share about ourselves and other people. So I guess with a connotation, where you kind of put ‘positive’ in front of a word, it doesn’t sit really well in a lot of practices that we do encourage in psychotherapy. – FGD 1

When you term it as positive it becomes very dichotomous, very off-putting… when [what] we want to talk is more about adaptability, workability, more neutral rather than there’s a negative or positive connotation. – FGD 1

They suggested alternative terms such as ‘mental wellness’, ‘positive living’, or sticking to words that were used by their clients, for instance ‘better life’ if that was what the client explicitly stated.

(3) Factors influencing PMH

Participants described several factors that could influence PMH and these were broadly classified into three categories: individual level, interpersonal level, and community and social cultural level. At the individual level, it was about clients’ personality and them having basic self-care which included things like exercise, proper sleep hygiene and healthy coping mechanism. For some participants, it was also about the clients having goals and purpose in life that could motivate them and which contribute to better wellbeing.

I think the other is having that sense of meaning and purpose, so feeling that I have meaningful visions, pursuits or meaningful job that I can contribute meaningfully to my system and the society at large. – FGD 3

At the interpersonal level, participants discussed interpersonal relationship with others that could influence PMH. This included support received from family, friends, or a significant other who provided the feeling of being ‘connected’ with others. A couple of participants noted the impact of mismatched values or misaligned expectations in relationships with others could have on the individuals.

But I think the other part is in the relationship with their significant other, the manner of how these values are transmitted or being talked about. Sometimes it can cause a lot of distress when they have different values. That’s where they have a lot of conflicts, especially when mental illness comes into the system which is a new thing, it can actually distraught the whole thing. – FGD 3

In terms of factors at the community level and social cultural level, a number of participants described how addressing stigma could be a step forward in improving PMH. One way to do so could be to reframe the idea of PMH:

But I was just wondering like why can’t PMH be same as growth and development so not assuming that you have a problem, but you just want to be resilient or be with some more resources. – FGD 4

Participants also suggested creating awareness and improving mental health literacy, particularly amongst the youth and within the school setting.

We are so driven by academic literacy that that’s pretty much all we know right, to achieve and strive, achieve and strive. And if we don’t get it then we fail. But there’s no emotional literacy and acceptance in that that is being taught in schools. – FGD 1

## Discussion

This was an exploratory study conducted to understand psychotherapists’ definitions of PMH and their views of this construct and its application in their clinical practice. From the findings reported in this study, it was observed that PMH was a multidimensional concept and while defined in varied manners, four main themes emerged from this qualitative inquiry. These themes identified are in many ways reflective of the conceptualizations of PMH and wellbeing in the current literature.

PMH in psychotherapy for the participants meant clients are able to alleviate distress and experience positive emotions. Considering that many of the study participants worked in clinical setting with clients who sought treatment for mood and anxiety disorders, it is expected that reducing distress would be a component described. This theme is in line with the hedonic traditions of mental health where the focus is on feeling well [23]. The hedonic approach also looks at life satisfaction which concurred with the theme on positive overall evaluation of life that was identified in this study. The theme on normal functioning and thriving in life identified in this study is reflective of the eudemonic viewpoint in which the focus is on functioning well psychologically and socially [24], and parallels could also be drawn with Ryff’s and Keyes’s concept of personal growth [5].

It was unclear at first glance if the theme on resilience accorded well with the hedonic and eudemonic traditions of conceptualizing PMH. A recent systematic review identified ‘growth’, ‘personal resources’ and ‘social resources’ as conceptualizations of resilience within adult mental health research [25]. In this sense it is comparable with Ryff’s and Keyes’s dimensions of personal growth and environmental mastery [5] where in the former individuals seeks development as a person, and in the latter being able to tap into individual and surrounding resources. Nevertheless, several authors have also suggested to include definitions of PMH that encompassed skills and coping strategies to achieve wellbeing [8, 26, 27]. Vaillant also proposed a cross-cultural definition of PMH that included viewing mental health as resilience [28]. Furthermore, this might be a pertinent concept for our study participants in the context of psychotherapy as clients are usually distressed and are seeking help to resolve their issues and return to normality, or to ‘bounce back’.

Results from this study showed that psychotherapists in our study, whose self-reported primary psychotherapeutic orientation was not amongst those in the fourth wave of psychotherapies (value- and virtue-oriented approaches such as positive psychology interventions, loving-kindness and compassion meditation and spiritually informed therapies; see [9]), generally see the value and potential in introducing PMH to their clients. However, PMH being novel and a ‘new science’ for some participants, unfamiliarity with it might act as a barrier for application in clinical settings. For one, some participants raised a point on the limitation of its use among clients presenting with more severe conditions. At this point, it might be worth highlighting that studies have been conducted among clinical samples which included patients with major depressive disorder and schizophrenia, and they provided preliminary evidence on the effectiveness of wellbeing interventions in improving wellbeing and reducing distress [11, 17, 29], with effects comparable with those of conventional cognitive behavioral therapy [30]. A qualitative study conducted among service users with psychosis to investigate their experience of positive psychotherapy also reported promising results. Feedback given was generally positive and participants provided instances of how the intervention supported them in making significant changes to their work and life domains [31]. In all, these studies lend support for the application of PMH interventions and incorporating them into psychotherapy practice.

Perhaps then the question to contemplate on is when or at which stage of therapy should interventions with elements of PMH be introduced to clients. Some therapists believed that while meaning in life is an underlying issue for all problems, it is not appropriate to address this with all clients in therapy. Client’s readiness and also presence of other pressing issues are factors to be considered [32]. In a similar vein, McNulty and Fincham noted that the effects of wellbeing traits and processes (e.g. optimism, positivity) are contextual based [33]; the interaction between a person’s characteristics and the social environment influences how these play out in either promoting or compromising wellbeing. Thus, psychotherapists would need to consider the circumstances in which to initiate PMH interventions, and future studies can seek to examine such factors that could potentially influence the effectiveness of these interventions.

Another finding worth discussing is that a number of participants were skeptical towards the use of the word ‘positive’. It is hard to discern if this reservation among our study participants is attributable to their background in clinical psychology and hence, the focus on deficits, or the unfamiliarity with PMH construct. The contention being that this terminology creates a dichotomy which is not an accurate nor ideal portrayal of mental health, and working towards a positive state all the time is not inherently desirable nor achievable. This echoes the argument by McNulty and Fincham that because psychological traits and processes have to be best understood in context [33], it would be prudent to avoid labeling them as positive or negative. However, as some authors have noted, these could be some common misconceptions surrounding positive psychological interventions [34, 35]. Rather, practitioners and researchers of PMH are advocating for a more balanced focus between illness and wellness. What this suggests is not replacing conventional psychotherapy modalities with PMH interventions, but instead complementing or supplementing the existing treatment options with them.

There are some limitations of this study to be noted. Firstly, some of the participants were acquainted with each other in the FGD session and that could potentially introduce participant bias in a way that their responses reflected the group’s sentiment rather than their own personal opinions. This was minimized by setting the scene from the beginning of the session where participants were explicitly informed that this was an exploratory study and there were no right or wrong answers to begin with. Participants were consistently asked if they agreed or disagreed with what was mentioned, and were encouraged to express their personal opinion in relation to the point raised. Secondly, the large majority of the participants were from public health institutions; only three participants were employed in private practice and one had experience in both. It is possible that differences in work practices and views exists between psychotherapists in public versus private setting, and this could limit the generalizability of the study findings.

## Conclusion

With the growing evidence and support for PMH and wellbeing interventions in the literature, it is an opportune time to explore service providers’ perspectives and views towards the use of these interventions in psychotherapy. This study found that the concept of PMH carried multiple meanings for psychotherapists in their practice that meant more than reduction of distress and alleviation of symptoms. It was generally agreed that PMH is an important concept and has a place in psychotherapy for clients, though some concerns may need to be addressed before it is introduced to them. Findings generated from this study provided valuable insights to understanding potential facilitators and barriers in integrating PMH interventions into psychotherapy.

## Supplementary Information

**Additional file 1.** COREQ Checklist.

## Data Availability

The datasets generated and/or analyzed during the current study are not publicly available due to requirements mandated by the institutional review board (IRB) and funders, but may be available from the corresponding author on reasonable request. Access may be granted subject to the IRB and the research collaborative agreement guidelines.
